# Phytochemical Profile and Antioxidant Activities of *Coleus amboinicus* Lour. Cultivated in Indonesia and Poland

**DOI:** 10.3390/molecules26102915

**Published:** 2021-05-14

**Authors:** Sylwester Ślusarczyk, Adam Cieślak, Yulianri Rizki Yanza, Małgorzata Szumacher-Strabel, Zora Varadyova, Marta Stafiniak, Dorota Wojnicz, Adam Matkowski

**Affiliations:** 1 Department of Pharmaceutical Biology and Botany, Wroclaw Medical University, 50-556 Wroclaw, Poland; marta.stafiniak@umed.wroc.pl; 2 Department of Animal Nutrition, Faculty of Veterinary Medicine and Animal Science, Poznań University of Life Sciences, 60-637 Poznan, Poland; adam.cieslak@up.poznan.pl (A.C.); yanza.pulspoznan@gmail.com (Y.R.Y.); malgorzata.szumacher@up.poznan.pl (M.S.-S.); 3 Centre of Biosciences of Slovak Academy of Sciences, Institute of Animal Physiology, 040 01 Košice, Slovakia; varadyz@saske.sk; 4 Department of Medical Biology and Parasitology, Wroclaw Medical University, 50-345 Wroclaw, Poland; dorota.wojnicz@umed.wroc.pl; 5 Botanical Garden of Medicinal Plants, Wroclaw Medical University, 51-601 Wroclaw, Poland

**Keywords:** *Coleus amboinicus*, *Plectranthus amboinicus*, Indian borage, rosmarinic acid, bioactivity, antioxidants, animal feed, diterpenoids, LC–MS, climatic zones

## Abstract

*Coleus amboinicus* Lour., *Lamiaceae*, is a perennial herb that is native to Indonesia and also cultivated in Africa, Asia and Australia. The major phytochemicals responsible for its bioactivity are rosmarinic acid (RA) and its analogues, flavonoids and abietane diterpenoids. The possibility of cultivation in a colder climate would extend the use of this herb and provide new opportunities to herb growers and livestock farmers. Our study to compare feed value and phytochemical composition of *C. amboinicus* plants cultivated in its original region, Indonesia, and in Poland. The crude protein content was significantly higher in plants cultivated in Indonesia compared to those cultivated in Poland—21% and 13% of dry matter, respectively. The higher ADF contents were detected in *C. amboinicus* cultivated in Indonesia, 38–41%, in comparison to 34% in plants cultivated in Poland. The phytochemical composition was also significantly influenced by the cultivation location. Polish samples were higher in polyphenols (RA and its analogues), and also had 1.5–2-fold higher antioxidant potential, as measured by DPPH scavenging, phosphomolybdenum reduction and Fenton reaction driven lipid peroxidation. The Indonesian samples contained more diterpenoid compounds, such as dihydroxyroyleanone, and the sum of terpenoids was ca. 10 times higher than in samples from Poland (15.59–23.64 vs. 1.87 µg/g of extracts). In conclusion, *C. amboinicus* is suitable for cultivation in non-optimal climatic conditions but some nutritional properties and bioactivity are significantly affected.

## 1. Introduction

*Coleus amboinicus* Lour. (synonym: *Plectranthus amboinicus* (Lour.) Spreng), *Lamiaceae*, is a perennial herb native to Indonesia and is widely cultivated in tropical Africa, Asia and Australia. *C. amboinicus* is used as a spice and ornamental plant as well as in folk medicine [1,2]. The species name, *amboinicus,* refers to Ambon Island in Indonesia, where it was apparently encountered. Ambon is part of the Maluku Islands of Indonesia [3]. In traditional medicine of India, the plant is used to treat a wide range of diseases: malaria fever, inflammation, cough, chronic asthma, bronchitis, liver diseases, renal and gallstones [3]. In Indonesia, it is used to stimulate lactation following childbirth, and also as an aromatic carminative and anthelmintic [4]. It is also used as a food additive and fodder. The leaves of the plant are often eaten raw or used as flavoring. In India, the leaves of *C. amboinicus* are consumed with buttermilk, yogurt, etc., during infection-induced diarrhea [5]. Over the years, several studies have been carried out confirming the very wide spectrum of *C. amboinicus* activity. In vivo studies showed that the plant has analgesic and anti-inflammatory activities [6], anti-inflammatory and antitumor activity against Sarcoma-180 and Ehrlich ascites carcinoma [7], antirheumatoid arthritis [8] and antimicrobial activity against *Staphylococcus aureus* [9]. In vitro studies also reported that the extract from *C. amboinicus* showed antioxidant and antibacterial activities [10,11], antifungal activity in food system [12] and antidandruff activity [13], as well as cytotoxic activity against breast cancer MCF-7 cells [14], and an antiproliferative effect against cancer cell lines: Caco-2, HCT-15, and MCF-7 [15]. Phytochemicals reported from this plant include a variety of phenolic compounds, such as several methoxylated flavonoids, hydroxycinnamic acids—rosmarinic acid, salvianolic acids A and L, shimobashiric acid C—and many minor constituents [16,17]. The second important phytochemical class in *C. amboinicus* are isoprenoids such as phytosterols [16], numerous volatile mono- and sesquiterpenoids in essential oils [1,18,19]. Recently, we have demonstrated the potential of *C. amboinicus* extract as a modulator of ruminal methanogenesis and biohydrogenation and suggested that a high content of rosmarinic acid is supposed to contribute to this effect. Other compounds that were present in the leaves extract included several flavone glycosides and diterpeneoid quinones, such as royleanone [20].

It needs to be highlighted that, regarding rumen metabolism, basic nutrient components such as crude protein and structural carbohydrates can interact with phytochemical components physically, decreasing their availability for rumen microbes [21]. Unfortunately, there is no research that would show unequivocally what compounds are formed during rumen metabolism. Recently, some changes in phytochemical components, i.e., saponins, have been shown in the ensiled material, where bacteria also play a significant role [22]; however, no such study has taken place on the rumen. Usually, indirect effects of the occurring processes are observed, e.g., on substrate digestibility, microbial populations, etc. The studies carried out in the rumen ecosystem are complex due to the omni-directional changes taking place there. It should be mentioned that concentration of phytochemical components also plays an important role in their detection. Due to the character of the rumen microbial ecosystem, more studies on the bioactive components bioavailability in ruminants are needed [23]. However, there is evidence that the bioavailability of phytochemical components such as polyphenols in feed where proteins or carbohydrates are the dominating nutrients depends on various factors such as: the structure of phenolics, microbial activity of enzymes or release of polyphenol from polyphenol—protein/polyphenol—carbohydrate complexes during ruminal microbial digestion [21,23]. On the other hand, there is evidence that polyphenols may also act as antinutritive components in ruminants feeding, inhibiting digestive enzymes, binding proteins or carbohydrates in feed, and finally reducing their ruminal degradation [24].

The above-mentioned effects, potentially beneficial for animals, drove us to consider introducing this plant to the region where the growth conditions are apparently unfavorable—i.e., the moderate, maritime influenced climate of West Poland (a region in Central Europe, Northern Hemisphere). As it was possible to grow these plants in the field during the vegetation season in Western Poland, we compared the LC–MS-based phytochemical profiles of *C. amboinicus,* cultivated in two plantations in Indonesia and in one plantation in Poland. Plants were cultivated under contrasting climatic conditions. Moreover, despite a few previous reports suggesting antioxidant properties of this plant, no compounds, nor a class of phytochemicals could be pinpointed as significantly contributing to its activity. Therefore, we have analyzed its antioxidant potential using three in vitro chemical models and developed a multivariate statistical comparison to assess which of the quantitatively determined or tentatively annotated phytochemicals contribute most to the activity. The nutritional value of the herbal material and digestibility in lamb feeding have been also evaluated.

## 2. Results

### 2.1. Phytochemical Analysis

Presently, only a few reports are available on the isolation and authentication of individual compounds from *C. amboinicus*, mainly concerning phenolic compounds. The information on these bioactive constituents was collected and discussed in a review over a decade ago [3]. Therefore, correct identification and quantification of phytocompounds with particular emphasis on the diterpene class (see Table 1) is necessary to understand their pharmacological and biological significance. In our previous experiments, phytochemical analysis showed that *C. amboinicus* extract contains phenolic acids (10.4 mg·g^−1^ dry matter), flavonoids (2.6 mg·g^−1^ DM), diterpenes (2 mg·g^−1^ DM), fatty acids (linolenic acid (35.4 g 100 g^−1^ fatty acids) and docosapentaonic acid (6.63 g 100 g^−1^ fatty acids) [25]. In the present study, the polyphenol contents (see Table 2) were the highest in extracts from leaves (CPL) and flowers (CPF) of plants grown in Poland: 112.95 ± 0.8 and 18.44 ± 0.6 mg·g^−1^, gallic acid equivalents, respectively. It was a markedly higher content than in plants grown under native climatic conditions: 23.61 ± 0.2 in (CI1) and 16.79 ± 1.5 mg·g^−1^ gallic acid equivalents (CI2). In turn, diterpenoid constituents were detected mainly in methanol extracts of plants grown in Indonesia (CI1) and (CI2) and formed a significant part of the all compounds in the extract, 23.64 ± 0.2 and 15.59 ± 0.2 mg·g^−1^ carnosic acid equivalents, respectively, whereas in samples grown in Poland, less than 1.0 mg·g^−1^ were found in all plant parts. Plants cultivated in Indonesia contained acetoxydihydroxyroyleanone as a major diterpene compound (16.64 mg·g^−1^ carnosic acid equivalents in CI1 and 10.17 mg·g^−1^ in CI2), followed by dihydroxyroyleanone (5.12 and 4.43 mg·g^−1^, carnosic acid equivalents, respectively). In plants from Poland, the amounts of these diterpenes were at negligible levels. The most abundant was rosmanol in the stems (CPS) (0.19 mg·g^−1^) and rosmadial in the leaves (CPL), flowers (CPF) and twigs (CPT) (0.8, 0.63 and 0.03 mg·g^−1^, carnosic acid equivalents, respectively).

### 2.2. Analysis of Plant Material

The significantly higher dry matter and crude fat were detected in CP1 compared to CI1 (Table 3). Crude protein content significantly differed between the plants grown in Indonesia (CI1 and CI2) and Poland (CP1). The CP1 has lower ADF content compared to CI1 and CI2, whereas aNDF concentration differed significantly between CP1 and CI2. Crude fat, ADF, and aNDF contents differed between plants from two Indonesian plantations. Regarding particular part of *C. amboinicus* cultivated in Poland, several differences were noticed (Table 3). Higher contents of organic matter, crude protein and crude fat were detected in CPF. The CPS was rich in ADF and aNDF, whereas CPL was rich in crude ash. A significantly higher crude protein intake was detected in CI2; however, the digestibility of all determined nutrients (dry matter, organic matter, crude protein, and aNDF) was similar in plants cultivated in Indonesia and Poland (Table 4).

### 2.3. Antioxidant Activity

The higher content of phenolic compounds in *C. ambonicus* Lour. cultivated in Poland was also clearly reflected in significantly higher antioxidant activity (Table 5) than in both Indonesian plantations.

### 2.4. Multivariate Analysis

The PCA score plot was used to present a natural correlation between the observations. To identify differential compounds, the Orthogonal Partial Least Squares Discriminant Analysis (OPLS-DA) model was used to explore differences in depth between profile metabolome of *Coleus* Indonesia and *Coleus* Poland samples. The OPLS-DA model with VIP values (VIP ≥ 1.0) and |p(corr)| ≥ 0.5 was selected as a differentiating compound.

**Figure 1:**
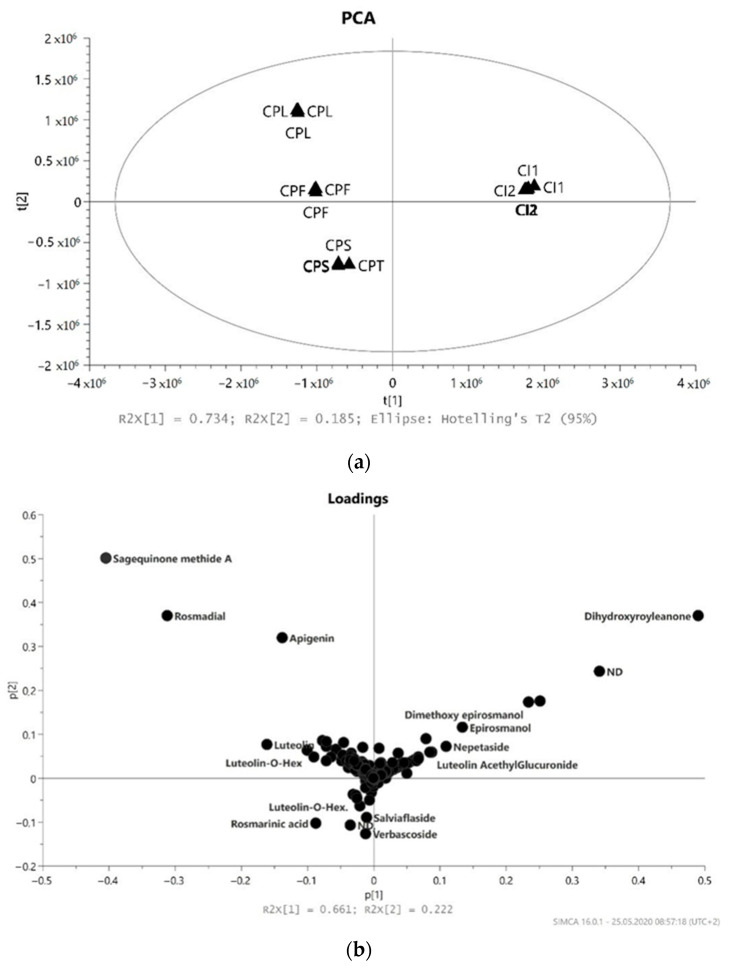

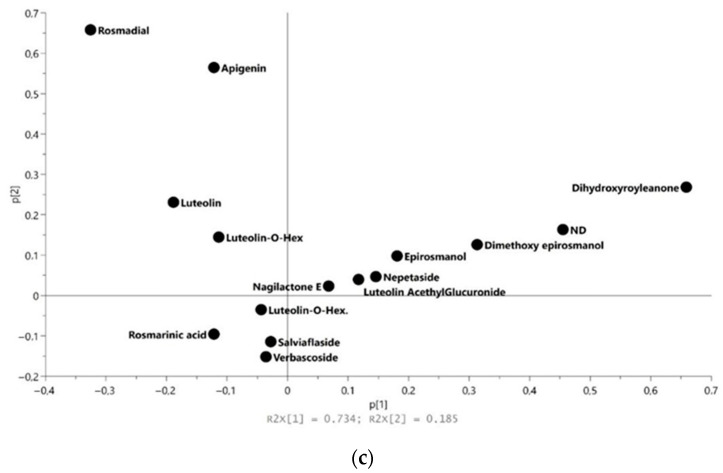
(**a**) PCA score plot based on UHPLC−MS data showing separation of *C. amboinicus* cultivated in Indonesia (CI1 and CI2) and leaves (CPL), flowers (CPF), stems (CPS) and twigs (CPT) from *C. amboinicus* cultivated in Poland with their respective 95% confidence regions. The explained variances are shown at the bottom; (**b**) the corresponding loadings scatter plot showing the compounds that are correlated to separation in scores plot; (**c**) the corresponding loadings scatter plot showing only the compounds that are correlated and most responsive to separation in scores plot.

**Figure 2:**
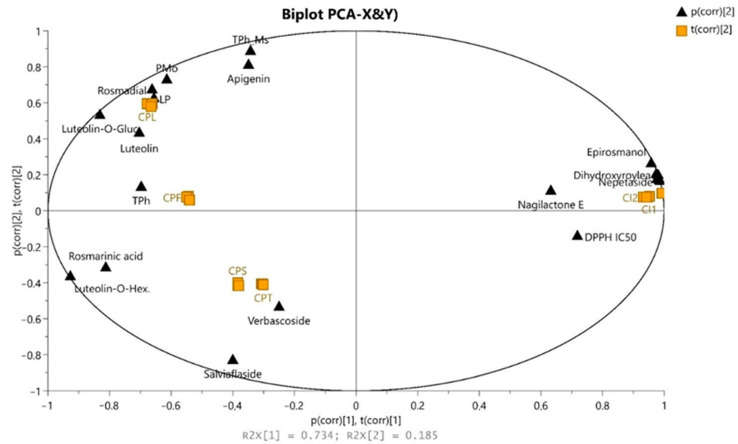
PCA biplot data showing correlation of *C. amboinicus* cultivated in Indonesia (CI1 and CI2) and leaves (CPL), flowers (CPF), stems (CPS) and twig (CPT) from *Coleus amboinicus* Lour. cultivated in Poland with their respective 95% confidence regions and antioxidant test.

It was also confirmed in principal component analysis (PCA). The first two principal components (PC1 and PC2) in the PCA model (Figure 1) cover almost 88.3% of the possible variation. The line score plot of the first principal components, PC1 vs. PC2, indicates a clear trend to form clusters (groups). The separation between samples from Indonesia and Poland was clearly observed. This might be due to the fact that the separation effect is associated with a complex of different compound classes. One of the distinguishable class of metabolites are flavonoids and phenolic acids in samples from Poland, and diterpenes (with acetoxy-dihydroxyroyleanone, dimethoxy-epirosmanol and dihydroxyroyleanone) in Indonesian samples. On the right side of score plots, the strong clusters, CI1 and CI2 (Indonesian samples), from both tested extracts were formed. This group is significantly distinguished from the other samples and strong negatively correlated with DPPH activity (EC_50_) according to loadings plot and positively correlated with high diterpenes accumulation (dihydroxyroyleanone, epirosmanol, rosmanol, acetoksy dihydroxyroyleanone). Additionally, low PMo assay activity in this group correlates with the lowest level of flavonoids and phenolic acids (Table 2). By using component 2 in plants cultivated in Poland (left side of score plots), we could discriminate three clusters: CPS, CPT together and separately CPL and CPF, which are positively correlated (loading plots) with phenolic compounds (TPh) mainly. Rosmarinic acid or flavonoids glucosides have less impact on differentiation in this group. All the extracts obtained from plants cultivated in Poland positively correlated with TPh content and all antioxidant tests. We can notice that the content of these compounds has the greatest impact on antioxidant activity. The score plots (Figure 2) indicate that CPS and CPT extracts are close to the center and have little influence on the first two principal components. The biplot simultaneously displays the relationship among scores and loadings. The scores (antioxidants assay and total polyphenols content) and loadings (variables important in projection (VIP) scores of 15 top contributors to PCA1 components) were expressed using correlation scaling. Observations of nearby variables are high, but are low in variables situated opposite.

**Figure 3:**
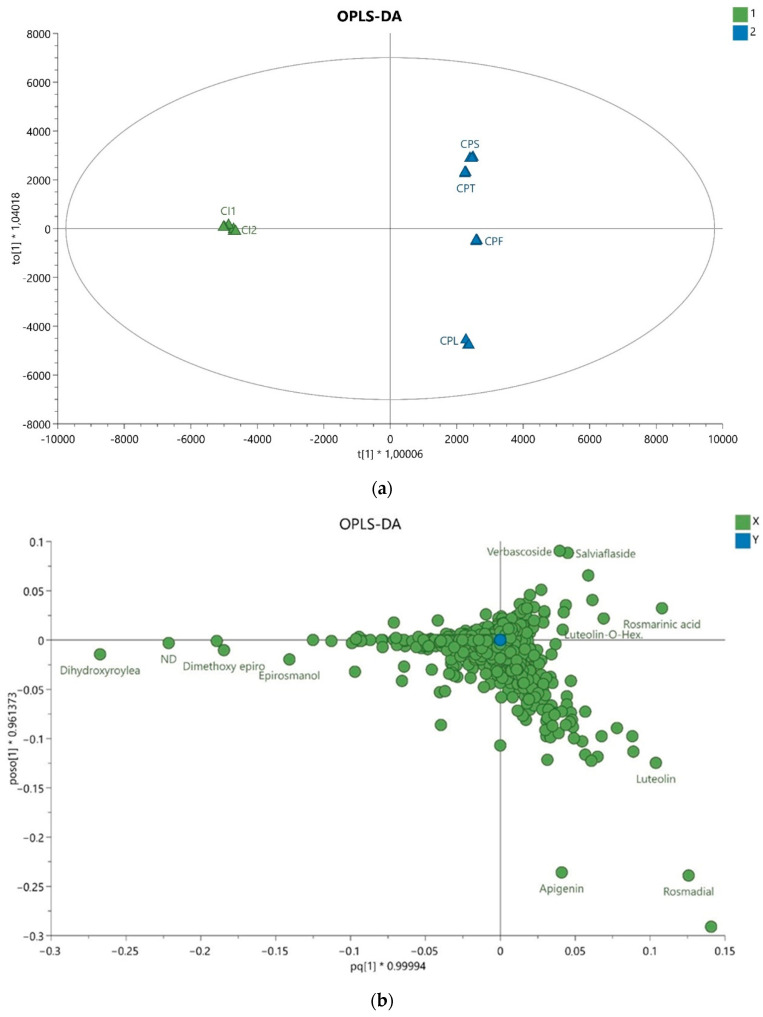
(**a**) Orthogonal Partial Least Squares Discriminant Analysis (OPLS-DA) score plot based on UHPLC−MS data showing separation of *C. amboinicus* cultivated in Indonesia (CI1 and CI2) and leaves (CPL), flowers (CPF), stems (CPS) and twig (CPT) from *C. amboinicus* cultivated in Poland with their respective 95% confidence regions. The explained variances are shown at the bottom; (**b**) the corresponding loadings scatter plot showing the compounds that are correlated to separation in scores plot.

OPLS-DA analysis (Figure 3) shows clear separation of clusters related to Indonesian and Polish samples, whereas the subdivision into different organs played a minor discriminative role, noticeable only for leaf samples vs. other parts. Diterpenoids are also confirmed as the major discrimination markers but flavonoids such as apigenin and some luteolin glycosides also have substantial positions.

## 3. Discussion

Differences in metabolic profiles and biological activity have been reported frequently in other plants collected or cultivated in distant regions [25]. For example, Rutkowska et al. [26] observed higher contents in several polyphenols (flavonol glycosides, chlorogenic acid, etc.) and stronger antioxidant properties of *Sorbus domestica* L. leaves from trees growing in Croatia than from Poland. However, the weather differences between those locations were not as distinct as in our case. The observations by Balabanova et al. [27] on metabolomic profile in *Portulaca oleracea* L. leaves indicated the importance of microclimatic and edaphic conditions in phytochemical diversity, including variation of phenolic and terpenoid compounds, but no clear clues about the individual factors were provided.

It can be supposed that the suboptimal climate caused an adaptive reaction in Polish plants by upregulating phenolic biosynthesis pathways at the expense of the diterpenoid (plastidic) pathway. Such responses to the climatic conditions are known in other plants [28,29]. The conditions differ dramatically between the tropical climate in Indonesia and the moderate Central European climate in terms of the periods of cold weather even during summers. However, the days are longer at higher latitudes, leading to longer insolation time during summer. The positive influence of longer days on the level of phenolic compounds has been reported for other plant species [25]. However, the total monthly solar irradiance is only slightly higher in Poland during first two months (June/July) of cultivation but significantly lower in August, which is consistent with the days becoming shorter. These results highlight the need of controlled illumination experiments to verify the day length and light intensity on the phenolic and terpenoid profiles in this plant.

On the other hand, temperature also affects metabolic profile. Elevated temperature can cause increase in both terpenoids and in phenolics, whereas lower temperatures are also known to stimulate phenolic accumulation [30,31]. The combination of temperature and higher solar irradiance may have led to earlier depletion of antioxidant compounds in Indonesian plants. The importance of combined climatic factors is of high relevance and of greater importance than the intensity of any individual parameter [32,33]. For example, de Medeiros Gomes [34], in an observational study on seasonal changes and *C. amboinicus’* phytochemical profile and antioxidant activity, reported significant fluctuations during the season, with a slight tendency for higher levels of rosmarinic acid and other polyphenols, with antioxidant activity correlating with summer months—i.e., higher solar irradiation. Unfortunately, terpenoids were not compared in their study.

In our study, however, the longer photoperiod with comparable ambient irradiation together with lower temperatures seems to be in favor of phenolics over terpenoid accumulation, resulting in higher antioxidant activity. Whether or not it was directly caused by this combination of environmental factors requires further study in controlled conditions. It also has to be borne in mind that mass spectrometry-based identification and chemometric analysis have important limitations. Firstly, the annotation of peaks is only fully confirmed when compared to authentic standards, which is usually a minor part of all detected compounds. Most of the compounds putatively identified from mass spectra are based on the literature and databases. It also hinders the absolute quantitation of the detected compounds that were relatively estimated using a few external standards—one for each major phytochemical class [35]. On the other hand, most of the detected compounds are not available for procurement and are present in minor amounts, which makes the isolation cost-inefficient. To overcome this, while still being able to use the data for relative comparisons between phytochemical profiles, one can use various approaches, such as fragmentation patterns [35,36,37,38,39,40], supported by a library search (used mainly in the present paper) or coupling (offline) to NMR spectroscopy [41]. The latter provides much more accurate structural information but is also less affordable and more laborious. The chromatography coupled with MS is widely adopted in GC–MS-based identification of volatile compounds or derivatized organics [37,42]. Yet, LC–MS-based metabolomics, despite huge progress, remains less standardized [42].

The LC–MS approach used in this study was based on the *m*/*z*-retention time pairs and normalized signals values to calculate the multivariate correlations and discrimination, an approach often used to compare plant samples within a taxon along with more advanced techniques [35,37,38,39,40], which allows compensating the lack of most phytochemical standards. Nonetheless, for absolute values, the standard-based quantification is advantageous.

*C. amboinicus* Lour. is known as a medicinal plant rich in crude protein and mineral components [1]. Plants from this species used in our study, can be also considered as a good source of protein—21% in plants cultivated in Indonesia and 13% in plants cultivated in Poland. *C. amboinicus* Lour. cultivated in Indonesia have the same level of protein as alfalfa meal (20–23% CP) [24]. In alfalfa, however, a negative linear relationship was found between crude protein and saponin content. In the present study, only other than saponins types of phytochemicals (phenolic acids, flavonoids, and diterpenoids) were determined, which can explain the lack of such correlations. For ruminants, NDF and ADF contents are important nutrients regarding scale of activity of rumen microorganisms. *C. amboinicus* cultivated in Indonesia (CI1 and CI2) contained higher levels of ADF than the Polish one (CP1); however, CI2 contained a higher level of NDF than the CI1 and CP1. In an in vitro study by Yanza et al. [20], *C. amboinicus* had medium contents of protein (196 g/kg DM) and lower contents of aNDF (363 g/kg DM) compared to the present study. We have concluded that cultivation of *C. amboinicus* under different climatic conditions and in various geographical locations gives more visible differences. Only crude protein and ADF contents prove this hypothesis. Other analysed basic nutrient components do not present the joined climatic and/or geographical patterns. This can partially explain the lack of differences in apparent total digestibility; however, crude protein intake differs between CI2 and CP1.

## 4. Materials and Methods

### 4.1. Plant Material Collection Characteristics and Preparation

*Coleus ambonicus* Lour., cultivated at Cianjur plantation (CI1), *Coleus ambonicus* Lour., cultivated in Bogor plantation (CI2) and *Coleus ambonicus* Lour., cultivated at Poznan plantation (CP1), were used in the study. The CI1 material was planted and randomly collected from different plots at the commercial farm located in Cianjur, West Java-Indonesia (6°43′30.1″ S 107°05′09.2″ E). CI2 material was planted and collected from the Karya Herbal Nasional Ltd. company land-plot at Bogor, Indonesia (6°70′28″ S, 106°99′90″ E). Both Indonesian plantations were grown on soil classified as Andosol (black volcanic), which are located near active volcanic mountains. The CP1 plants were grown in Poznan, Poland, at experimental field on soil classified as mold, located at Wielkopolska, Poznan, Poland (52°22′15″ N 17°42′40″ E). In Indonesia, CI1 and CI2 were grown under tropical climate (from 16 to 29 °C) with intensive rainfalls and high humidity. The CP1 was grown under a moderate climate with average temperatures during the growing season (June–August) from 19 to 24 °C. The solar irradiance intensity 10 years average during June–August for both locations are following (according to Solar Radiation interactive tool of the European commission’s Photovoltaic Geographical Information System, https://ec.europa.eu/jrc/en/pvgis, accessed on 1 May 2021)—June 147.5 ± 11.3 vs. 162.1 ± 14.9, July 163.3 ± 16.8 vs. 166.4 ± 15.6, August 178.0 ± 19.6 vs. 141.6 ± 14.6 kWh/m^2^, in Cianjur and Poznań, respectively.

During the cultivation, CP1 plants were regularly irrigated. The Indonesian plant material (leaves) was harvested and collected after 3 months of cultivation, dried at 50 °C in the bedding oven for 2 days, then grounded in the milling machine. The dried Indonesian plant material was packaged and shipped to the Department of Animal Nutrition Poznan University of Life Sciences, Poland. The same procedure was applied for preparing samples from plants cultivated in Poznan, Poland. Additionally, the plants from Poland were divided into leaves (CPL), flowers (CPF), main stem (CPS) and lateral branches, and twigs (CPT).

### 4.2. Analysis of Plant Material

Samples of plant material were analyzed following the procedures of AOAC [43] for dry matter (DM; method no. 934.01), crude ash (method no. 942.05), crude protein (CP; using a Kjel-Foss Automatic 16,210 analyzer; method no. 976.05), and ether extract (EE; using a Soxhlet System HT analyzer; method no. 973.18) concentrations [30]. Organic matter (OM) was calculated by subtracting ash concentration from DM content. Ash-free neutral detergent fiber (aNDFom) was determined following Van Soest et al.’s method with addition of amylase and sodium sulfide without residual ash [44].

### 4.3. Phytochemical Analysis

Determination of the polyphenolics and diterpenes content performed using Liquid chromatography–High-Resolution Mass Spectrometry (LC–HRMS).

About 100 mg of each materials were ground to a fine powder and extracted three times with 80% (*v*/*v*) MeOH at 40 °C for 60 min, then the obtained extracts were combined and evaporated to dryness. In total, 20 mg of each extract were dissolved in 3 mL of Milli-Q water (acidified with 0.2% formic acid) and purified by Solid Phase Extraction (SPE) using Oasis HLB 12 cc 500 mg Vac Cartridge (Waters Corp., Milford, MA, USA). The cartridges were washed with 0.5% methanol to remove carbohydrates and washed with 80% methanol to elute analytes of lower polarity. The obtained fraction was re-evaporated and dissolved (2.5 mg) in 1 mL of 80% methanol (acidified with 0.2% formic acid). Samples were centrifuged (23,000× *g*, 5 min) and stored in a freezer at −30 °C until analysis.

The analytical system consisted of a Dionex UltiMate 3000RS (Thermo Scientific, Darmstadt, Germany) system with DAD detector interfaced via electrospray ionization module with a high-resolution quadrupole time-of-flight mass spectrometer (HR/Q-TOF/MS, Compact, Bruker Daltonik GmbH, Bremen, Germany). Separation was performed using a Kinetex C18 column (2.1 × 100 mm, 2.6 μm, Phenomenex, Torrance, CA, USA), with mobile phase A consisting of 0.1% (*v*/*v*) FA in water and mobile phase B consisting of 0.1% (*v*/*v*) FA in acetonitrile. A linear gradient from 7% to 50% phase B in phase A over 20 min was used to separate phenolic compounds with a short 0.5 min equilibration segment. The sample injection volume was 5 µL, the flow rate was 0.3 mL/min and the column was held at 25 °C. Spectra were acquired in negative- and positive-ion mode over a mass range from *m*/*z* 100 to 1500 with 5 Hz frequency. Operating parameters of the ESI ion source were as follows: capillary voltage—3 kV, dry gas flow—6 L/min, dry gas temperature—200 °C, nebulizer pressure—0.7 bar, collision radio frequency—700.0 V, transfer time—100.0 μs, and pre-pulse storage—7.0 μs. Ultrapure nitrogen was used as the drying and nebulizer gas and argon was used as the collision gas. Collision energy was set automatically from 15 to 75 eVi depending on the *m*/*z* of fragmented ions. Acquired data were calibrated internally with sodium formate introduced to the ion source at the beginning of each separation via 20 μL loop. Processing of spectra was performed with Bruker DataAnalysis 4.3 software (Bruker Daltonik GmbH, Bremen, Germany).

Annotation of individual metabolites was based on their retention times and mass spectral data obtained in negative mode with those of standard compounds or with compounds previously reported in the literature for Coleus species [1,20,45]. A tentative identification of phenolics and diterpenes was performed based on key fragment ions and other MS observations. For flavonoids and their glycosyl derivatives, the loss of 176 *m*/*z* was indicative of oxyhexosyl (glucurononyl), 162 *m*/*z* was indicative of hexose (glucose or galactose), the loss of 146 *m*/*z* was indicative of deohexosyl (rhamnose), the loss of 132 *m*/*z* was indicative of pentose (xylose or arabinose). Moreover, for diterpenes and flavonoids, the loss of 42 *m*/*z* (acylate) 44 *m*/*z* in the negative-ion mode and the loss of 86 *m*/*z* were indicative of the presence of a malonate. For this purpose, the available Human Metabolome Database and MetFragWeb databases (with KEGG, PubChem, ChemSpider databases) were used. The proposed fragmentation pathway with main daughter ion and fragments are shown in (Table 1). A comparison of individual metabolites was made of their retention times and mass spectral data obtained in negative mode with those of standard compounds or with compounds previously reported [20]. The amount of the individual phenolic acids in an extract were calculated as rosmarinic acid (CAS 537-15-5 (*R*)-rosmarinic acid, EDQM, Strasbourg, Europe) equivalent and isoquercitrin (CAS 482-35-9 Quercetin 3-*O*-glucopyranoside, Sigma-Aldrich, St. Louis, MO, USA) was used for the calculation of identified flavonoids. Stock solutions of rosmarinic acid and isoquercitrin were prepared in MeOH at concentrations of 3.2 and 4.5 mg/mL, respectively, and kept frozen until used. Calibration curves for these two compounds were constructed based on seven concentration points (from 500 to 3.9 µg/mL). The amount of the diterpenes were calculated as carnosic acid (CAS 3650-09-7, Supelco, Bellefonte, PA, USA) equivalent on seven concentration points (from 125 to 0.05 µg/mL).

The linearity for the calibration curves for carnosic acid, isoquercitrin and rosmarinic acid each had R^2^ ≥ 0.998. The DAD detector was set to three different wavelengths—254, 280, and 330 nm. Consequently, the peak areas measured at 280 nm were used to calculate the concentrations of the analytes for carnosic acid, at 320 nm for rosmarinic acid, and 254 nm for isoquercitrin. All analyses were performed in triplicates.

### 4.4. Total Polyphenols Determination by the Folin–Ciocalteau Method

A method based on our previous paper was applied [36]. To 40 mL of extract (1 mg/mL), 3.16 mL of deionized water was added, followed by 200 mL of Folin–Ciocalteau reagent (POCh, Gliwice, Poland). After 5 min, 600 mL of 20% (*w*/*w*) sodium carbonate solution was added. For the blank, 40 μL of water was used instead of the extract. Control tests were performed for all extracts, in which the Folin–Ciocalteau reagent has been replaced with an appropriate amount of deionized water. The prepared solutions were left at room temperature in a dark place for 2 h. After this time, 300 μL were taken into 96-well plate from each sample and absorbance at 765 nm was measured. The results were expressed as gallic acid equivalents (GAEs).

### 4.5. DPPH Assay

Quenching of a nitrogen-based synthetic free radical—diphenyl-2-picrylhydrazyl (DPPH, Sigma-Aldrich, St. Louis, MO, USA)—was performed as in our previous study [46]. The following starting concentrations of the tested extracts in 80% methanol were prepared: 500, 200, 100, 50, 20, 10, 4, 2 µg/mL. Then, 125 µL of each solution was taken into the 96-well plate and 125 µL of about 200 µmol DPPH was added. The absorbance of the resulting solutions was measured spectrophotometrically at 517 nm, every 3 min for 30 min. A mixture of 125 mL 90% methanol with an equal volume of DPPH solution was used as a control, while ascorbic acid and quercetin solutions were used as positive controls. For each dilution of the tested extracts, measurements were performed in 3 replicates, from which the results were averaged. Based on the obtained means, the EC_50_ of all extracts for DPPH elimination was calculated.

### 4.6. Phosphomolybdenum Assay

The measurement of the total antioxidant capacity by the phosphomolybdenum test is a method based on the reduction of Mo (VI) to Mo (V) by the compounds contained in the tested sample [36,47]. The reaction proceeds in an acidic environment to form green-blue complexes of reduced phosphomolybdate that are measured spectrophotometrically at 695 nm. For samples of unknown composition, measured properties antioxidants can be expressed as equivalents of tocopherol, ascorbic acid or another adequate antioxidant. Graphs of concentration dependence of absorbance were prepared for the quercetin and ascorbic acid as tested samples. Slope coefficients of simple linear functions, K1—straight slope coefficient for vitamin C, K2—straight slope coefficient for the test sample/quercetin, were calculated. Then, the K2/K1 ratio was determined, illustrating the reduction potential of the tested extracts relative to the ascorbic acid standard (% ascorbic acid equivalents—AAEs).

### 4.7. Linoleic acid Peroxidation Assays

The ability to inhibit reactive oxygen species-mediated oxidation of polyunsaturated lipids was tested in a linoleic acid-Fe-H_2_O_2_ model (Fenton type reaction) similarly to our previous papers [46]. Briefly, the extract at 50 µg/mL (150 μL) was mixed with 1050 μL of 0.1 M/pH 7.4 phosphate buffer-linoleic acid emulsion (linoleic acid mixed with Tween 80, 3:1, *w*/*w*); then, the emulsion/extract mixture was transferred to 5 mL of 0.2 mM mL ascorbic acid in 0.1 M phosphate buffer (pH 7.4). The peroxidation was started with the addition of 150 μL 10 mM FeSO_4_ and incubated for 90 min at 37 °C. Thereafter, 1.5 mL of 10% ice-cold trichloroacetic acid and 1.5 mL of 1% thiobarbituric acid in 50 mM NaOH were added and heated in a water bath at 90 °C for 10 min. After cooling the samples and mixing with 2 mL of *n*-BuOH, the absorbance was read at 532 nm after transferring 300 μL of the BuOH phase from samples to the 96-well plate. Test results are expressed as % inhibition of linoleic acid peroxidation.

### 4.8. Determination of Feed Intake and Total Track Digestibility of Lambs

The experimental procedure used at this stage of the study was performed in accordance with the guidelines of the National Ethical Commission for Animal Research (Ministry of Science and Higher Education, Poland). The study was approved by the Local Ethical Commission (license permission no. 35/2019). The eight growing lambs (20 ± 3 kg live weight) were used for the feed intake and total digestibility determination. Animals were randomly allocated into CI2 or CP1 dietary groups (*n* = 4). CI2 was chosen due to the numerically highest crude protein content. Lambs were kept individually to record daily feed intake and amount of faces. Animals had free access to drinking water. The experiment lasted 30 days: a 21-day adaptation stage and an 8-day sampling period. The lambs in CI2 and CP1 were fed with grass silage (400 g/day; 41.6% of DM; 18.7% of CP in DM; 45.6% of aNDF in DM) and concentrate mixture (400 g/day; 88.9% of DM; 20.3% of CP in DML; 23.8% of aNDF in100 g DM). Additionally, each feeding group received 200 g/d of dry powder of CI2 or CP1 per day. The chemical compositions of CI2 and CP1 are presented in Table 2.

The apparent total tract digestibility (TTD) of DM, OM, CP, and aNDF were calculated based on the following equation:

$$\mathrm{TTD}=\frac{{\mathrm{intake}\;\mathrm{of}\;\mathrm{DM}\;\mathrm{or}\;\mathrm{OM}\;\mathrm{or}\;\mathrm{CP}\;\mathrm{or}\;\mathrm{aNDF}}_{\frac{g}{\mathrm{day}}\;}-{\mathrm{outputs}\;\mathrm{of}\;\mathrm{DM}\;\mathrm{or}\;\mathrm{OM}\;\mathrm{or}\;\mathrm{CP}\;\mathrm{or}\;\mathrm{aNDF}}_{\frac{g}{\mathrm{day}}}}{{\mathrm{intake}\;\mathrm{of}\;\mathrm{DM}\;\mathrm{or}\;\mathrm{OMor}\;\mathrm{CP}\;\mathrm{or}\;\mathrm{aNDF}}_{\frac{g}{\mathrm{day}}}}$$

### 4.9. Statistical Calculations

Calculations of statistical parameters and differences between results for individual samples were made in GraphPad Prism 8.0.1. The comparison of means obtained from DPPH and Linoleic acid peroxidation assays were performed by variance analysis using one-way ANOVA with post hoc tests and Student’s *t*-test for evaluation of the statistical significance of the results (results not shown). Results from all assays were collected by three independent experiments with three repetitions of each data point. For all of these analyses, a *p* value ≤ 0.05 was considered to be statistically significant. A one-way analysis of variance (ANOVA) and Tukey’s post hoc test were also carried out assuming the statistical significance of differences for *p* ≤ 0.05.

#### Data Processing and Multivariate Analysis (PCA)

Profile Analysis software (version 2.3, Bruker Daltonik GmbH, Germany) was used to preprocess the raw UHPLC−QTOF-MS data. Profile Analysis parameters were set as follows: advanced bucket generation with retention time range of 1.0−25.0 min, mass range of 100−800 *m*/*z*, without normalization, with background subtraction and time alignment. LC−MS analyses were processed with the Find Molecular Futures (FMF) function to create compounds (molecular features) with signal-to-noise threshold of 3 for peak detection. Generated bucket table consisting of *m*/*z*−retention time pairs and respective compound intensity were imported into Metabolists 4.0 (http://www.MetaboAnalyst.ca/, accessed on 31 January 2021) online software to estimate missing values and to filter and normalize data (normalization by median). No transformation was generalized and data matrix was mean-centered and divided by the square root of the standard deviation of each variable (Pareto scaling). Then, the obtained data matrix was introduced into SIMCA-P+16.01 (Umetrics, Umeå, Sweden) software for in review multivariate statistical analysis of principal component analysis (PCA). The PCA score plot was used to present a natural correlation between the observations. To identify differential compounds, the OPLS-DA (Orthogonal Partial Least Squares Discriminant Analysis) model was used to explore differences in depth between the phytochemical profile of Indonesian and Polish Coleus samples. The OPLS-DA model with VIP values (VIP ≥ 1.0) and |p(corr)| ≥ 0.5 was selected as a differential compound. This provides a preliminary overview of features that are potentially significant for the separation of the two groups (plants cultivated in Poland and Indonesia).

## 5. Conclusions

In conclusion, *C. amboinicus* Lour. is suitable for cultivation in non-optimal climatic conditions that would extend the use of this herb and provide new opportunities to herb growers and livestock farmers. The nutrient contents, i.e., crude protein and/or ADF, depend on the cultivation conditions. Some quality parameters were inferior but higher antioxidant activity and polyphenol content suggest a good potential for using *C. amboinicus* Lour. in animal nutrition. Optimization of agronomic measures to eliminate the negative environmental impacts is also required.

## Figures and Tables

**Table 1:** The most abundant, tentatively identified compounds in the *Coleus amboinicus* samples from Indonesia (CI1, CI2) and Poland (CPL, CPF, CPS, CPT); their contents were estimated using carnosic acid (for diterpenoids), rosmarinic acid (for phenol carboxylic acids) and isoquercitrin (for flavonoids) evaluation (mg·g^−1^ extract, *n* = 3, mean value).

Peak	RT(min)	λ_max_ (nm)	Molecular Ion *m*/*z* [M-H]^−^	MS^2^ Main Ion	MS^2^ Fragments	Formula	Tentative Identification	Phenolic Acids	Flavonoid	Diterpenes	References
**CI1**											
1	1.3	281	197.0447	135.0442	179,151,123	C_9_H_10_O_5_	Syringic acid	0.17			HMDB0002085
2	1.5		167.0335	123.0446	149	C_8_H_8_O_4_	Vanillic acid	0.09			HMDB0000484
3	1.6		153.0175	135.0423	123	C_7_H_6_O_4_	Dihydroxybenzoic acid	0.12			HMDB0013676-7
4	2.5		137.0232	93.034		C_7_H_6_O_3_	Hydroxybenzoic acid	1.35			HMDB0002466
5	4.7	242,322	179.0347	135.0441		C_9_H_8_O_4_	Caffeic acid	3.91			HMDB0001964
6	6.9		371.0992	249.0614	174,121	C_16_H_20_O_10_	Dihydroferulic acid-O-Glu	0.32			HMDB0041723
7	8.5		345.1550	183.1072	165,208	C_16_H_26_O_8_	Nepetaside				HMDB0038149
8	10.4	342	447.0942	285.0411	174	C_21_H_20_O_11_	Luteolin-O-Hex		0.5		HMDB0035588
9	10.6	255,348	461.0746	285.0407		C_21_H_18_O_12_	Luteolin-O-Glucur		4.8		HMDB0240541
10	11.0		193.0504	161.0235	178,134	C_10_H_10_O_4_	Ferulic acid	0.33			HMDB0000954
11	11.2	288,330	359.0777	161.0231	197,179,135	C_18_H_16_O_8_	Rosmarinic acid derivative	0.34			HMDB0003572
12	11.5	267,335	445.0779	269.0459		C_21_H_18_O_11_	Apigenin-O-Hex		4.07		HMDB0240480
13	11.6	288,329	359.0777	161.0231	197,179,135	C_18_H_16_O_8_	Rosmarinic acid	3.05			standard
14	12.1	254,349	503.085	285.0409	163	C_23_H_20_O_13_	Luteolin-O-(Glucur-Maloyl)		2.11		HMDB0041384
15	13.1	268,332	589.121	269.0458	427,161	C_27_H_26_O_15_	Apigenin derivative		1.24		a
16	13.6		493.2445	331.1916	161,221		Carnosic acid derivative			0.11	standard
17	13.9		285.0412			C_15_H_10_O_6_	Luteolin		0.19		HMDB0005800
18	14.3		593.1298	447.0947	285	C_30_H_27_O_13_	Luteolin-O-dHex-Hex		0.16		a
19	15.7		269.0462	225.0567	151	C_15_H_10_O_5_	Apigenin		0.18		HMDB0002124
20	16.0	220,322	329.067	314.0441	299,285	C_17_H_14_O_7_	3′,7′-Dimethylquercetin		0.11		HMDB0029263
21	16.6	220,317	491.0989	179.0346	293,267,135,161	C_26_H_20_O_10_	Salvianolic acid C	0.31			a
22	16.9	219,310	329.1763	285.1863	227,189,171	C_20_H_26_O_4_	Diterpene derivative			0.45	a
23	17.1		491.0979	179.0346	267,311,135,161	C_26_H_20_O_10_	Salvianolic acid C derivative	0.15			a
24	17.9		313.0721	298.0479	283,269	C_17_H_14_O_6_	5,7-Dihydroxy-4′,6-dimethoxyflavone		0.11		HMDB0128589
25	18.7		333.2079	289.2182	271,179	C_20_H_30_O_4_	Dihydroxykaurenoic acid			0.03	HMDB0036760
26	19.5		351.2177	305.2115		C_20_H_32_O_5_	Trihydroxy-*ent*-kauranoic acid			0.04	HMDB0036756
27	20.3	288	345.1713	327.1602	301,283,208,317	C_20_H_26_O_5_	Rosmanol			0.11	HMDB0036661
28	21.0		333.2076	289.2182	271,219,165	C_20_H_30_O_4_	Dihydroxykaurenoic acid			0.19	HMDB0036760
29	22.3	218,274	347.1868	317.1762	299	C_20_H_28_O_5_	Nagilactone E			0.49	a
30	22.5	218,274	347.1875	329.1768	319	C_20_H_28_O_5_	Dihydroxyroyleanone			5.12	a
31	23.4		345.1719	315.1612	283.1714,301	C_20_H_26_O_5_	Epirosmanol			0.13	HMDB0035812
32	23.8		333.1717	289.1821	261,271,245,306	C_20_H_30_O_4_	Dihydroxy-16-kauren-19-oic acid			0.11	HMDB0036763
33	24.0		343.1559	328.1319	315,300,287,271	C_20_H_24_O_5_	a Diterpene			0.22	a
34	24.4	273	389.1985	347.1878	329,311,301,285,	C_22_H_30_O_6_	Acetoxy-dihydroxyroyleanone			16.64	a
**CI2**											
1	1.3	281	197.0447	135	179,151,123	C_9_H_10_O_5_	Syringic acid	0.35			
2	1.5		167.0335	123.0446	149	C_8_H_8_O_4_	Vanillic acid	0.03			
3	1.6		153.0175	135.0423	123	C_7_H_6_O_4_	Dihydroxybenzoic acid	0.25			
4	2.5		137.0232	93.034		C_7_H_6_O_3_	Hydroxybenzoic acid	0.7			
5	4.7	242,322	179.0347	135.0441		C_9_H_8_O_4_	Caffeic acid	2.48			
6	6.9		371.0992	249.0614	174,121	C_16_H_20_O_10_	Dihydroferulic acid-O-Glucur	0.18			HMDB0041723
7	8.5		345.1550	183.1072	165,208	C_16_H_26_O_8_	Nepetaside				
8	10.4	342	447.0942	285.0411	174	C_21_H_20_O_11_	Luteolin-O-Hex		0.33		
9	10.6	255,348	461.0746	285.0407		C_21_H_18_O_12_	Luteolin-O-Glucur		3.88		
10	11.0		193.0504	161.0235	178,134	C_10_H_10_O_4_	Ferulic acid	0.17			
11	11.2	288,330	359.0777	161.0231	197,179,135	C_18_H_16_O_8_	Rosmarinic acid derivative	0.33			
12	11.5	267,335	445.0779	269.0459		C_21_H_18_O_11_	Apigenin-O-OHex		1.7		
13	11.6	288,329	359.0777	161.0231	197,179,135	C_18_H_16_O_8_	Rosmarinic acid	3.66			
14	12.1	254,349	503.0850	285.0409	163	C_23_H_20_O_13_	Luteolin-O-(Glucur-Maloyl)		1.34		
15	13.1	268,332	589.1210	269.0458	427,161	C_27_H_26_O_15_	Apigenin derivative		0.51		
16	13.6		493.2445	331.1916	161,221		Carnoscic acid glucoside			0.05	
17	13.9		285.0412			C_15_H_10_O_6_	Luteolin		0.16		
18	14.3		593.1298	447.0947	285.0401	C_30_H_27_O_13_	Luteolin-O-Dhex-Hex		0.14		
19	15.7		269.0462	225.0567	151	C_15_H_10_O_5_	Apigenin		0.06		
20	16.0	220,322	329.0670	314.0441	299,285	C_17_H_14_O_7_	3′,7′-Dimethylquercetin		0.16		HMDB0029263
21	16.6	220,317	491.0989	179.0346	267,311,135,161	C_26_H_20_O_10_	Salvianolic acid C	0.22			
22	16.9	219,310	329.1763	285.1863	227,189,171	C_20_H_26_O_4_	Diterpene derivative			0.28	
23	17.1		491.0979	179.0346	267,311,135,161	C_26_H_20_O_10_	Salvianolic acid C derivative	0.08			
24	17.9		313.0721	298.0479	283,269	C_17_H_14_O_6_	5,7-Dihydroxy-4′,6-dimethoxyflavone		0.04		
25	18.7		333.2079	289.2182	271,179	C_20_H_30_O_4_	Dihydroxykaurenoic acid			0.02	
26	19.5		351.2177	305.2115		C_20_H_32_O_5_	Trihydroxy-ent-kauranoic acid			0	
27	20.3	288	345.1713	327.1602	283,208,317	C_20_H_26_O_5_	Rosmanol			0.06	
28	21.0		333.2076	289.2182	271,219,165	C_20_H_30_O_4_	Dihydroxykaurenoic acid			0.14	
29	22.3	218,274	347.1868	317.1762	299	C_20_H_28_O_5_	Nagilactone E			0.06	
30	22.5	218,274	347.1875	329.1768	319	C_20_H_28_O_5_	Dihydroxyroyleanone			4.43	
31	23.4		345.1719	315.1612		C_20_H_26_O_5_	Epirosmanol			0.08	
32	23.8		333.1717	289.1821	261,271,245,306	C_20_H_30_O_4_	Dihydroxy-16-kauren-19-oic acid			0.09	
33	24.0	289	343.1559	328.1319	315,300,287,271	C_20_H_24_O_5_	Diterpene			0.21	
34	24.4	273	389.1985	347.1878	329,311,301,285,	C_22_H_30_O_6_	Acetoksy dihydroxyroyleanone			10.17	
**CPL**											
1	1.3	281	197.0453	135.0435	179,151,123	C_9_H_10_O_5_	Syringic acid	0.33			
2	1.5		167.0335	123.0446	149	C_8_H_8_O_4_	Vanillic acid	0.04			
3	1.6		153.0176	123.0447		C_7_H_6_O_4_	Dihydroxybenzoic acid	0.65			
4	4.7	242,322	179.0345	135.0437		C_9_H_8_O_4_	Caffeic acid	3.48			
5	9.5	255,342	447.0929	285.0405	174	C_21_H_20_O_11_	Luteolin-O-Hex		9.81		
6	9.5	274	509.2391	347.1874			ND		1.14		
7	10.3	255,348	461.0730	285.0406		C_21_H_18_O_12_	Luteolin-O-Glucur		12.27		
8	11.5	267,337	445.0778	269.0462	175	C_21_H_18_O_11_	Apigenin-Glucur		17.59		HMDB0240480
9	11.6	286,329	359.0781	161.0234	179,197,135	C_18_H_16_O_8_	Rosmarinic acid	11.35			
10	12.0	254,347	503.0830	285.0408		C_23_H_20_O_13_	Luteolin-O-(Glucur-Maloyl)		5.28		
11	12.2		421.2074	289.1663	233,161	C_19_H_34_O_10_	Octen-3-yl-beta-primeveroside			0.53	HMDB0032960
12	12.7	267,336	487.0875	383.0771	269,311	C_24_H_24_O_11_	Apigenin-O-(maloyl-Pentosyl)		4.54		a
13	13.3		487.0882	269.0461		C_24_H_24_O_11_	Apigenin-O-acetylglucuronide		20.05		a
14	13.4		503.0824	443.0619	285.0406	C_23_H_20_O_13_	Luteolin 3′-(3-acetylglucuronide)		1.85		HMDB0038808
15	13.9	345	285.0409			C_15_H_10_O_6_	Luteolin		12.67		
16	14.7	267,337	487.0882	269.0461	427	C_24_H_24_O_11_	Apigenin-O-acetylglucuronide		7.02		
17	14.9	286	343.1557	325.1453	310,295	C_20_H_24_O_5_	Rosmadial			0.8	HMDB0038219
18	15.0		271.0609	151.0023		C_15_H_12_O_5_	Trihydroxyflavanone		0.18		HMDB0031824
19	15.6	267,336	269.0466	225.056	151.0023	C_15_H_10_O_5_	Apigenin		3.9		
20	16.0	335	313.0728	161.0233		C_17_H_14_O_6_	ND		0.53		
21	16.4	289	343.1546	325.1449	310,295	C_20_H_24_O_4_	Diterpene			0.54	
22	16.8		491.0982	267.066	311,179,161,135	C_26_H_20_O_10_	Isosalvianolic acid	0.27			
**CPF**											
1	0.8		191.0195	129.0174		C_6_H_8_O_7_	Citric acid	0.26			HMDB0000094
2	1.0		299.0775	137.0229	164	C_13_H_16_O_8_	Salicylic acid beta-D-glucoside	0.19			HMDB0041271
3	1.3	281	197.0453	135.0435	179,151,123	C_9_H_10_O_5_	Ethyl gallate	0.9			HMDB0033836
4	1.5		167.0335	123.0446	149	C_8_H_8_O_4_	Vanillic acid	0.07			
5	1.6		153.0547	123.0447		C_7_H_6_O_4_	Dihydroxybenzoic acid	0.37			
6	4.7	242,322	179.0345	135.0437		C_9_H_8_O_4_	Caffeic acid	1.39			
7	9.5	262,342	447.0929	285.0405	174	C_21_H_20_O_11_	Luteolin-O-Hex		7.05		
8	10.4	266,348	461.0725	285.0405		C_21_H_18_O_12_	Luteolin-O-Glucur		3.46		
9	10.5		436.2242	316.1676	290,145	C_25_H_31_N_3_O_4_	Dicoumaroylspermidine	0.97			
10	10.6		431.0973	269.0455	311	C_21_H_20_O_10_	Apigenin 7-O-beta-D-glucoside		0.45		
11	11.0		461.1085	299.056		C_22_H_22_O_11_	Kaempferide 7-glucoside		0.14		HMDB0038455
12	11.1		475.0874	285.04	447	C_22_H_20_O_12_	Luteolin 4′-methyl ether 7-glucuronide		0.07		
13	11.5		445.0773	269.0455	285,175	C_21_H_18_O_11_	Apigenin 7-glucuronide		1.96		
14	11.6		359.0781	161.0234	179,197,135	C_18_H_16_O_8_	Rosmarinic acid				
15	12.1		503.0833	285.0408	343,161	C_23_H_20_O_13_	Luteolin-O-(Glucur-Maloyl)				
16	12.2		507.2234	345.1707	327.1606,489	C_26_H_36_O_10_	Rosmanol-hexosyl			0.2	
17	13.9	345	285.0409			C_15_H_10_O_6_	Luteolin		0.45		
18	14.5		509.2394	347.1876	329,301	C_30_H_38_O_7_	ND	0.08			
19	15.0		343.1561	310.122	325,295	C_20_H_24_O_5_	Rosmadial			0.63	
20	15.7	329	269.0457	151.0022	225	C_15_H_10_O_5_	3,4′,7-Trihydroxyflavone		0.63		
**CPS**											
1	1.3	281	197.0453	135.0435	179,151,123	C_9_H_10_O_5_	Syringic acid	0.07			
2	1.5		167.0335	123.0446	149	C_8_H_8_O_4_	Vanillic acid	0.13			
3	1.6		153.0175	135.0423	123	C_7_H_6_O_4_	Dihydroxybenzoic acid	0.19			
4	2.1		341.0879	179.0339	161,135	C_15_H_18_O_9_	1-O-Caffeoylglucose	0.19			HMDB0036937
5	2.5		137.0232			C_7_H_6_O_3_	Hydroxybenzoic acid	0.12			
6	4.7	242,322	179.0342	135.0437		C_9_H_8_O_4_	Caffeic acid	0.88			
7	9.5		447.0942	285.0411	174	C21H20O11	Luteolin-O-Hex		1.12		
8	9.7		537.1033	295.061	313	C_27_H_22_O_12_	Lithospermic acid	0.06			a
9	9.8		521.1287	359.0787	265,161,135	C_24_H_26_O_13_	Salviaflaside deriv	0.09			
10	10.2	288,331	623.1981	461.1662	161,315,179	C_29_H_36_O_15_	Verbascoside	0.36			HMDB0034843
11	10.3		461.0730	285.0406		C_21_H_18_O_12_	Luteolin-O-Glucur		1.04		
12	10.4		521.1301	359.0829	323,197,161	C_24_H_26_O_13_	Salviaflaside	0.45			
13	10.5		477.1405	323.0781	161,179	C_23_H_26_O_11_	Calceolarioside A	0.26			a
14	10.6		521.1298	359.0989	197,161,179	C_24_H_26_O_13_	Salviaflaside	0.21			HMDB0033705
15	10.9	288,331	623.1977	461.1662	161,315,179,135	C_29_H_36_O_15_	Isoverbascosode	0.07			HMDB0041025
16	11.0		461.1086	299.056	285,341	C_22_H_22_O_11_	Kaempferide 7-glucoside		0.11		
17	11.1		193.0501	161.0229	178,135	C_10_H_10_O_4_	Methyl caffeate	0.09			a
18	11.2		475.0879	285.0409	406,31	C_22_H_20_O_12_	ND		0.59		
19	11.6		359.0781	161.0234	179,197,135	C_18_H_16_O_8_	Rosmarinic acid	9.2			
20	11.9		665.2095	461.166	161	C_31_H_38_O_16_	Tubuloside B			0.11	a
21	12.7		537.1032	295.0614	161,197,135,359	C_27_H_22_O_12_	Melitric acid	0.13			HMDB0040680
22	12.8		717.1460	321.0402	339,295,185,515	C_36_H_30_O_16_	Salvianolic acid L	0.14			HMDB0037370
23	13.0		673.3084	510.2454	348	C_32_H_50_O_15_	ND	0.14			
24	13.3		503.0819	285.0402	443,367,218	C_23_H_20_O_13_	Luteolin 3′-(4″-acetylglucuronide)		0.11		
25	13.9	345	285.0409			C_15_H_10_O_6_	Luteolin		0.72		
26	15.6	329	269.0460	151.0022	225	C_15_H_10_O_5_	3,4′,7-Trihydroxyflavone		0.08		
27	19.4		345.1709	327.1604	301,309,294	C_20_H_26_O_5_	Rosmanol			0.19	
28	20.2		345.1709	327.1604	301,317,303	C_20_H_26_O_5_	Epirosmanol			0.05	
29	20.9		329.1767	311.1664	285	C_20_H_26_O_4_	Carnosol			0.03	HMDB0002121
30	22.1		487.3424	469.3303		C_30_H_48_O_5_	Madasiatic acid derivative			0.01	HMDB0035118
31	22.6		347.1875	329.1768	319	C_20_H_28_O_6_	Dihydroxyroyleanone			0.02	
**CPT**											
1	0.8		191.0195	129.0174	154	C_6_H_8_O_7_	Citric acid	0.33			
2	1.3	281	197.0453	135.0435	179,151,123	C_9_H_10_O_5_	Syringic acid	0.27			
3	1.5		167.0335	123.0446	149,137	C_8_H_8_O_4_	Vanillic acid	0.01			
4	1.6		153.0536	123.0444		C_7_H_6_O_4_	Dihydroxybenzoic acid	0.15			
5	4.7	242,322	179.0345	135.0434		C_9_H_8_O_4_	Caffeic acid	0.65			
6	9.5	262,342	447.0941	285.041	174	C_21_H_20_O_11_	Luteolin-O-Hex		1.85		
7	10.2	285,321	328.1195	161.0227	175,149,133		ND		0.38		
8	10.3	266,348	461.0725	285.0405		C_21_H_18_O_12_	Luteolin-O-Glucur		0.68		
9	10.5		521.1301	359.0857	323,161,197,179	C_24_H_26_O_13_	Salviaflaside deriv	0.24			
10	10.7	285,323	521.1302	359.0858	323,161,197,180	C_24_H_26_O_13_	Salviaflaside	0.18			
11	11.0	342	461.1087	299.0563	341	C_22_H_22_O_11_	Kaempferide 7-glucoside		0.09		
12	11.1	334	475.0883	285.0408	447,406	C_22_H_20_O_12_	Luteolin 4′-methyl ether 7-glucuronide		0.07		
13	11.5		445.0773	269.0455	285,175	C_21_H_18_O_11_	Apigenin 7-glucuronide		0.2		
14	11.6		359.0781	161.0234	179,197,135	C_18_H_16_O_8_	Rosmarinic acid	3.47			
15	11.9	339	475.0887	299.0559		C_22_H_20_O_12_	Kaempferide 7-glucuronide		0.04		
16	12.1	334	503.0833	285.0408	343,161	C_23_H_20_O_13_	Luteolin-O-(Glucur-Maloyl)		0.16		
17	13.4	334	503.0817	285.0408	443,399	C_23_H_20_O_13_	Luteolin-O-(Glucur-Maloyl) isomer		0.05		
18	13.9	345	285.0409			C_15_H_10_O_6_	Luteolin		0.07		
19	14.6		361.1661	343.1557	299,333,317	C_20_H_26_O_6_	Diterpene			0.04	
20	15.0		343.1551	310.1212	325,295	C_20_H_24_O_5_	Rosmadial			0.03	
21	15.6		551.2493	329.176	301,283		Diterpene derivative			0.05	

ND—not determined; Hex—hexosyl (glucosyl, galactosyl); dHex—deoxyhexosyl; Glu—(glucosyl); Glucur—(glucuronyl, galacturonyl), HMDB ID—The Human Metabolome Database; MetFrag—MetFrag Online DataBase.

**Table 2:** Phytochemical components present in *Coleus amboinicus* Lour. cultivated in Indonesia (CI1 and CI2) and in Poland (CPL, CPF, CPS, CPT) (mg·1 g^−1^ Extract, *n* = 3, mean value ± SD, as gallic acid equivalents (for total phenols), carnosic acid equivalents (for diterpenoids), rosmarinic acid equivalents (for phenol carboxylic acids) and isoquercitrin equivalents (for flavonoids) based on UHPLC−MS data.

Sample (mg·1 g^−1^ Extract)	CI1	CI2	CPL	CPF	CPS	CPT
Total phenols	23.61 ± 0.2	16.79 ± 1.5	112.95 ± 0.8	18.44 ± 0.6	16.55 ± 0.9	8.89 ± 0.5
Phenolic acids	10.14 ± 1.2	8.45 ± 0.8	16.12 ± 1.3	4.23 ± 0.4	12.78 ± 1.1	10.6 ± 1.3
Flavonoids	13.47 ± 0.9	8.34 ± 0.6	96.83 ± 1.4	14.21 ± 1.1	3.77 ± 0.2	7.18 ± 1.1
Diterpenes	23.64 ± 0.2	15.59 ± 0.2	1.87 ± 0.1	0.83 ± 0.04	0.41 ± 0.02	0.24 ± 0.08

**Table 3:** Primary chemicals content in *Coleus amboinicus* Lour. cultivated in Indonesia (CI1 and CI2) and in Poland (CP1) samples) and separately sample from leaves (CPL), flowers (CPF), stems (CPS), and twigs (CPT) of plants cultivated in Poland (*n* = 3, mean value ± SD). Values marked with the same superscript letters in rows, are not significantly different at *p* ≤ 0.01.

Nutritional Component (g/100 gDM)	CI1	CI2	CP1	*p* Value	CPL	CPF	CPS	CPT	*p* Value
Dry matter	90.4 ± 0.04 ^b^	92.8 ± 0.64 ^ab^	94.2 ± 0.30 ^a^	<0.01	93.8 ± 0.55	94.2 ± 0.08	94.8 ± 0.04	94.5 ± 0.04	0.56
Organic matter	85.5 ± 0.00	84.4 ± 0.11	85.0 ± 3.46	0.83	83.2 ± 0.11 ^b^	90.1 ± 0.10 ^a^	85.7 ± 0.05 ^ab^	86.3 ± 0.16 ^ab^	0.01
Crude ash	14.5 ± 0.00	15.6 ± 0.11	15.0 ± 3.34	0.81	16.8 ± 0.11 ^a^	9.86 ± 0.01 ^b^	14.3 ± 0.05 ^a^	13.7 ± 0.16 ^a^	<0.01
Crude protein	21.1 ± 0.08 ^a^	21.4 ± 0.39 ^a^	13.3 ± 5.32 ^b^	0.03	15.9 ± 0.14 ^b^	18.6 ± 0.44 ^a^	7.50 ± 0.13 ^c^	10.6 ± 0.32 ^c^	<0.01
Crude fat	4.13 ± 0.01 ^b^	4.56 ± 0.13 ^a^	4.52 ± 0.05 ^a^	0.01	3.04 ± 0.11 ^b^	4.27 ± 0.06 ^a^	0.86 ± 0.09 ^c^	1.13 ± 0.02 ^c^	0.01
ADF	40.8 ± 0.47 ^a^	37.9 ± 0.26 ^b^	34.0 ± 0.01 ^c^	<0.01	26.5 ± 2.08 ^c^	30.8 ± 0.14 ^c^	43.3 ± 0.92 ^a^	38.2 ± 0.06 ^b^	<0.01
aNDF	39.1 ± 0.43 ^b^	42.1 ± 0.76 ^a^	40.6 ± 0.03 ^b^	0.01	33.6 ± 0.18 ^c^	36.6 ± 0.55 ^c^	54.0 ± 0.41 ^a^	43.8 + 0.38 ^b^	0.01

CI1: *Coleus ambonicus* Lour. cultivated at Cianjur plantation; CI2: *Coleus ambonicus* Lour. cultivated at Bogor plantation; CP1: *Coleus ambonicus* Lour. cultivated at Poznan plantation; CPL: leaves; CPF: flowers; CPS: main stems; CPT: lateral branch twigs; DM: dry matter; aNDF: ash Neutral Detergent Fiber.

**Table 4:** Comparisons of feed intake and total track digestibility of lambs fed *Coleus amboinicus* Lour. (*n* = 4, mean value).

Parameter	CI2	CP1	*p* Value
Feed intake (g/d)			
Dry matter	807.3 ± 47.4	787.3 ± 68.7	0.35
Organic matter	683.2 ± 42.5	668.0 ± 63.7	0.44
Crude protein	122.3 ± 4.9	107.2 ± 3.8	<0.01
aNDF	206.2 ± 41.1	224.3 ± 30.7	0.20
Digestibility			
Dry matter	0.64 ± 0.45	0.65 ± 0.53	0.66
Organic matter	0.63 ± 0.46	0.64 ± 0.57	0.65
Crude protein	0.58 ± 0.44	0.57 ± 0.59	0.44
aNDF	0.36 ± 0.77	0.38 ± 0.11	0.57

CI2: *Coleus ambonicus* Lour. cultivated at Bogor plantation; CP1: *Coleus ambonicus* Lour. cultivated at Poznan plantation; DM: dry matter; ADF: acid digestible fiber, aNDF: ash Nutral detergent fiber.

**Table 5:** Comparisons of feed intake and total track digestibility of lambs fed with *Coleus amboinicus* Lour. (*n* = 3, mean value).

Parameter	CI1	CI2	CPL	CPF	CPS	CPT
DPPH EC_50_ (µg/mL)	95.46 ± 1.2	152.8 ± 1.6	60.69 ± 1.3	32.67 ± 0.2	114.6 ± 0.2	57.53 ± 0.1
PMo (% AAE)	7.062 ± 0.7	9.806 ± 0.1	16.95 ± 0.2	14.37 ± 0.1	8.267 ± 0.3	9.57 ± 0.1
LP (50 µg/mL)	39.66 ± 1.1	32.69 ± 0.3	78.22 ± 0.6	69.27 ± 1.2	53.35 ± 0.4	28.42 ± 0.2
TPh (mg GAE/g)	22.22 ± 0.4	25.56 ± 0.2	57.89 ± 2.2	98.89 ± 1.1	30.22 ± 0.2	61.22 ± 3.2
Phenolics UPLC (mg·1 g^−1^)	23.61 ± 0.2	16.79 ± 1.5	112.95 ± 0.8	18.44 ± 0.6	16.55 ± 1.1	8.89 ± 0.7
Diterpenes UPLC (mg·1 g^−1^)	23.64 ± 0.2	15.59 ± 0.2	1.87 ± 0.2	0.83 ± 0.2	0.41 ± 0.2	0.24 ± 0.2

DPPH—diphenyl-2-picrylhydrazyl free radical EC_50_ of all extracts for DPPH elimination, quercetin was used as reference with EC_50_ at 4.6 µg/mL; PMo—phosphomolybdenum test, value expressed as % of ascorbic acid equivalents; LP—Linoleic acid peroxidation assay, results are expressed as % inhibition of linoleic acid peroxidation in relation to the control sample without any antioxidant; TPh—total polyphenol by Folin–Ciocalteu colorimetric method, results expressed as gallic acid equivalents.

## Data Availability

Data are contained within the manuscript.

## Sample Availability

Samples of the plants extract are available from the first author (S.Ś.).
